# Notes and News

**Published:** 1898-04-09

**Authors:** 


### NOTES AND NEWS.

(Collected and Communicated.)

The North Eastern Hospital for Children is appealing for £15,000 to extend its accommodation.

The Quarantine Council at Cairo has decided to apply the plague regulations against arrivals from Jeddah.

The festival dinner on behalf of Earlswood Asylum is to be held on Friday, May 20th, at the Hotel Cecil, when the Duke of Leeds has promised to preside.

The Lord Mayor is receiving at the Mansion House contributions to the Home of Rest memorial to the Duchess of Teck.

A series of lectures on tropical medicine is to be held during the summer session at the Middlesex Hospital, and Deputy Surgeon-General H. Cayley, F.R.C.S., has been appointed to deliver the course.

The probability of war has led over 300 physicians to volunteer as acting assistant-surgeons in the United States Navy, but these offers cannot be accepted until Congress authorises such temporary appointments. It is pointed out by the New York *Medical Record* that, notwithstanding all this rush for temporary employment, there are already about twenty vacancies in the Regular Service, for which there are few or no applications, because of the humiliating treatment to which newly-appointed assistant-surgeons are subjected. The question would appear to take much the same form in America as in England, although the difficulty affects the Navy on one side of the Atlantic and the Army on the other. Clearly, it is neither the work, the hardship, nor the danger that keeps men out of the service; nor, indeed, is it the money question. The difficulty is a social one. The best medical students used to be drawn to the Army by exactly the same feelings as influence those who join the combatant branches of the service, viz., by a hope of reward for good service and of passing their lives in a confraternity of officers and gentlemen. Deny them the latter and the whole glamour is wiped away, and then they not unnaturally prefer independence and private practice.

The Prince of Wales's Hospital Fund has received £500 from the Corporation of London, this sum representing the current year's instalment of the £5,000 voted by this body.

We regret to have to record the death of Surgeon-Captain E. Corcoran from fever at Okuta, in the Hinterland of Lagos, upon March 23rd. The Army Medical Staff thus loses a promising officer, who has done good service with the troops in West Africa since September last. Surgeon-Captain Corcoran's commission bore date February 5th, 1887. Whether in peace or war, the medical officers of the British army are always exposing themselves to danger at the call of duty.

One of the race problems that has lately forced itself upon the attention of dwellers in the United States is the excessive death-rate which prevails among the negroes in the cities of the South. It appears that in the following five cities, Atalanta, Baltimore, Charleston, Memphis, and Richmond, the average death-rate of the whites during the 15 years ending in 1895 was 21 per 1,000, while the rate among the coloured was 36 per 1,000. The reason given for this is interesting, it being stated that the first cause of this high mortality is the excessive number of coloured women who go out to work, and have thus to neglect their children. Only one coloured man in four, it appears, supports his family without assistance. The inherent laziness of the male savage is evidently at the root of the matter, for it must take many generations to knock out of the descendants of the African his tendency to depend on the labour of his women. Female labour in tropical Africa did not matter, the women had their children with them; but female labour in towns means the separation of mother and child, and this—as we have found out at this side also—is the root of much evil.

In the presence of a considerable outbreak of small-pox at Middlesbrough, in England, and as a comparison with what is being done by the sanitary authorities for the relief of the sufferers in that town, it is interesting to note what is going on in the town of Middlesboro', in Kentucky, where there is also an outbreak of small-pox. According to the *Medical News* of New York, 40 cases of variola and 29 suspects are there quarantined in the pest-house, and there are no funds to pay for their care! Dr. McCormack, Chief Inspector of the State Board of Health, says the State has no funds to be used for the purpose, the county refuses to make an appropriation, and the city is bankrupt. Federal assistance can be had, but only on the invitation of the State Board of Health, which, however, objects to Federal intervention. There is clearly something political at the back of all this, about which we know nothing. Meanwhile, the Chief Inspector of the State Board of Health threatens that if the county does not render the necessary aid he will release the patients. The latter are said to have been two days without food, and to be threatening to make their escape. We have often heard of quarrels between public authorities in England, but we have not yet come across anything quite equal to this in the way of one authority bringing pressure to bear upon another.

